# Users’ perceptions of how an unmoderated eating disorder community may benefit or harm their recovery

**DOI:** 10.1186/s40337-022-00653-3

**Published:** 2022-08-31

**Authors:** Maegan E. Jones, Elizabeth H. Blodgett Salafia, Kerrie C. Leonard

**Affiliations:** 1 Department of Psychology, St. Cloud State University, 103 Stewart Hall, 720 Fourth Ave. S., St. Cloud, MN 56301-4498 USA; 2 Human Development and Family Science, North Dakota State University, Fargo, USA

**Keywords:** Eating disorders, Social media, Qualitative, Recovery, Motivation

## Abstract

**Background:**

While the negative and positive outcomes of pro-eating disorder groups have been well-documented, more research is needed on the effects of pro-recovery communities. In particular, a gap in knowledge remains surrounding users’ feelings about their experiences in such communities. Using open-ended questions, we surveyed those involved in the recovery community on the social media site Tumblr, to determine how the community helped/did not help with recovery, and how it made them feel about recovery.

**Methods:**

Participants (*n* = 29) answered a series of Likert and open-ended questions. The open-ended questions were examined using thematic analysis to learn about users’ lived experiences.

**Results:**

Themes identified included connection/acceptance, motivation/encouragement, content, and competition/comparisons. Participants overall felt that the community helped them feel connected to others and accepted for their struggles and their successes, though a minority did note that such acceptance could sometimes reinforce negative behaviors, and connection could lead to pressure to help others. Participants found both direct advice and indirect support (e.g., encouraging posts) to be motivating for their recovery. Feelings of competition and comparison were mixed; some felt that comparing to others made them feel less than, while others used such comparisons as reminders to stay strong in recovery. Participants indicated that the content within the community varies; sometimes there is a large presence of pro eating disorder posts, while at other times, posts are more positive, motivational, and encouraging.

**Conclusions:**

Participants overall indicated that the community had many helpful aspects and made them feel better about recovery. However, the presence of triggering content and competition in particular were notable drawbacks of the community. Overall, recovery communities on unmoderated sites or social media applications may be useful tools during the recovery process, despite some important limitations.

## Plain English summary

Recovery communities on Tumblr and other social media sites are not moderated by a trained eating disorder professional, but are often an important source of connection for those going through treatment, or those unable to currently access professional treatment. This study asked Tumblr users who participated in the eating disorder recovery community about their experiences, and specifically what they thought about how the community might help or harm their recovery. Users felt the community was overall beneficial, and that the community helped users feel connected, motivated, and encouraged. However, they felt the community could also be triggering and competitive, and comparisons to others in recovery could be helpful or harmful, depending on the context. This information is important for understanding how unmoderated social media sites might support those as they continue on their eating disorder recovery journeys.

## Background

There is a plethora of research on the negative impacts of pro-eating disorder (ED) communities on the internet, such as increased restriction and increased identification with one’s ED (e.g., [11]). Yet, they are also sources of emotional support, connection, and understanding for users (e.g., [9, 16, 20]). A limited number of studies have examined moderated, pro-recovery online environments and found similar positive patterns; such sites empowered users to not feel alone in their struggles, feel safe in expressing thoughts and feelings, create friendships, and find support [1, 14].

However, what about *unmoderated* social media communities, where professionals are not present? Such communities remain understudied, even though a substantial minority of ED-related posts are pro-recovery [4]. One study found that positive encouragement from peers on Facebook was associated with less ED pathology, while comparison was related to increased symptomology [17]. Similarly, recent examinations of recovery communities on Reddit and TikTok found that users shared recovery experiences and successes, emphasized the importance of connection, and more, though some tagged “recovery” content was identified as pro-ED [6, 13]. Therefore, unmoderated pro-recovery communities may also positively and negatively impact users.

While such research has added to the field’s knowledge regarding unmoderated pro-recovery communities, it is also essential to understand lived experiences within such groups. Indeed, Branley and Covey [4] noted a need to determine how ED content is experienced, and how that affects behaviors and thoughts. One study that interviewed participants about their usage of ED sites noted that, while it was sometimes difficult to avoid triggering content, most who engaged in unmoderated pro-recovery communities did not report negative experiences [19]. However, pro-recovery posts on blogs have more negative emotionality compared to pro-ED blogs, as users acknowledge the harmful feelings that led to their ED [22]. Therefore, it is important to explore how individuals feel about shared content within communities, and how potentially negative emotional expressions from others might affect their recovery.

Two theoretical frameworks guided the present study. Egosyntonic refers to the harmony or alignment of one’s thoughts, feelings, and behaviors, to their ego; egosyntonic theory applies to EDs, Anorexia Nervosa (AN) in particular. According to this theory, identity is intertwined with EDs, and individuals with AN value their disorder and it provides a source of identity [12, 21]. Symptoms characteristic of AN such as extreme weight loss may not be perceived as a problem and are instead viewed as achievements [21]. Thus, the egosyntonic aspect of AN is a barrier to treatment because individuals may not be motivated to recover [12]. Perhaps then engaging in Tumblr does not provide benefit in treatment for those who do not acknowledge the harm.

The transtheoretical model of change (TTM) is another theoretical framework which relates to motivations in treatment. The TTM is a stage theory proposing that individuals progress through six stages of readiness to change: precontemplation, contemplation, preparation, action, and maintenance [15]. In terms of eating disorders, an individual in the precontemplation stage does not want to seek recovery, while an individual in the contemplation stage is considering pursuing recovery. Cockell and colleagues [10] examined women with AN in either of these two stages and found that those who were seriously contemplating change endorsed more costs to the disorder and had more insight in how AN provides functional avoidance (i.e., escapism). These results suggest that knowledge of the harmful effects can aid in one’s motivation to pursue recovery. Tumblr may provide a space for education for its users and motivate individuals with EDs to recover. Those in the precontemplation stage may also endorse more egosyntonic views of their identity, and struggle more with triggering content. Conversely, those in the contemplation, preparation, action, and maintenance phases may identify less with their eating disorder symptoms and more with their recovery identity.

Unmoderated communities will continue to proliferate as social media use becomes a seemingly integral part of everyday life (e.g., [3]). Those in ED recovery have also reported increased feelings of social isolation due to the COVID-19 pandemic, which negatively affected their eating habits [5]; unmoderated recovery communities may provide an important avenue for reconnection. More research is needed to determine precisely how unmoderated, pro-recovery environments affect users’ experiences, and which aspects may encourage growth versus undesirable feelings about the self. Specifically, this study will examine the recovery community on one website, Tumblr; we aimed to determine which facets of the community helped users’ recovery, and how the community made users feel about recovery.

## Method

According to the about section of Tumblr.com at the time of study creation, Tumblr users can, “post texts, photos, quotes, links, music, and videos from your browser, phone, desktop, email or wherever you happen to be.” Users also have a “dashboard” where posts from other blogs appear; users can comment, reblog, and add hashtags. Tumblr has an “ask” feature, where users can send messages, both as themselves and anonymously. There is also a direct messaging feature.

Tumblr was chosen because the setup of the site mimics more traditional “blog” sites, while combining the features of newer social media sites (e.g., videos, hashtags), giving users a variety of ways to engage. We also believed this site allowed us to be authentic about our identities as researchers with a science-based background who believe recovery is possible, without encouraging users to see us as a recovery resource.

An account was created for our lab after IRB approval. With the exception of the survey post, we did not create any content, only reblogging informational posts or motivational posts already on Tumblr. Informational posts were chosen based on our positionality as scientific researchers who believe it is important to combat misinformation and stereotypes. We did not interact with any users; the messaging feature was disabled. A secure Qualtrics link was shared in a post, using recovery-related hashtags such as #EDrecovery, #eatingdisorderrecovery, and #EDtreatment. International recovery resources were listed at the top of each survey page, as was a reminder that participants could skip questions. A combination of 18 Likert-style and open response questions were included in the survey (see “Appendix 1”). Questions were developed by the first author, and the second author provided feedback. Questions were then piloted with two research assistants, who made suggestions for further clarification.

The initial survey page asked if users were above the age of 13; if participants indicated they were not, they were unable to continue the survey. If they indicated they were 13 or older, they were taken to a consent page, where the potential risks and benefits of the study were explained using language appropriate for youth assent. The IRB approved a waiver for the acquisition of parental consent, as it was not possible to contact users' parents through Tumblr. No personal or identifying information was collected, and we did not ask questions about participants’ EDs beyond diagnosis, in order to minimize potential distress.

For this paper, five of the questions were examined using theoretical thematic analysis at a semantic level [7]. These questions were chosen for these analyses in order to focus on the participants’ lived experiences related to helpful and harmful recovery experiences on Tumblr, and how those experiences may affect their own recovery [4]. The first question asked, “Does the recovery community on Tumblr help you with your eating disorder recovery?” with response options including no, a little, sometimes, often, and most of the time. Two open-ended questions then followed, asking, “What about the recovery community helps your eating disorder recovery?” and “What about the recovery community do you think makes your eating disorder recovery harder?” Finally, the open-ended question, “After viewing eating disorder recovery posts on Tumblr, do you usually feel better or worse about your own recovery?” was asked, with a follow-up open-ended question of “Why or why not?” The Likert-style question results are also available in “Appendix 2” for examination.

The six phases of thematic analysis as described by Braun and Clarke [7] were followed. After reading the responses several times, the first author generated initial codes related to positive and negative experiences within the community, searched for and reviewed themes, and defined and named themes cultivated from the data related to support/motivation, connection, comparison, and content. While the original analysis was completed on a question by question basis, upon reviewer feedback, themes were reanalyzed based on the dataset as a whole. The first author initially engaged in this process. For the first analysis, the second author and a research assistant checked codes and reviewed themes. The third author originally participated in defining and naming themes, and during the reexamination process, checked the first authors’ interpretation of codes and themes across the dataset. The second author acted as a critical peer when discrepancies arose. This process produced a more coherent set of themes, minimizing redundancies. All authors participated in producing the report. Participants’ responses have not been edited, to stay true to their voices.

## Results

### Participants

Participants who answered at least half of the questions (29 out of 31) were included in the analyses. Participants ranged in age from 15 to 43, (M = 20.71, SD = 7.04), were majority White (*n* = 26), female (*n* = 26), and indicated having an ED diagnosis from a doctor (*n* = 25). Of those that had an official diagnosis, seventeen listed Anorexia Nervosa (AN), three Eating Disorder Not Otherwise Specified/Other Specified Feeding or Eating Disorder (EDNOS/OSFED), three Bulimia Nervosa (BN), one Binge Eating Disorder (BED), and one did not answer. Of those that stated they had not been officially diagnosed, one indicated they had BN, while another believed they had EDNOS. Of the 27 participants that responded to a question about professional help-seeking, 21 (77.8%) indicated they were receiving professional aid, while 6 (22.2%) indicated they were not.

All but one participant felt that the recovery community helped at least a little with their recovery. Results were more mixed when participants were asked if they felt better or worse about their recovery after viewing content, with five participants noting they felt worse, seven stating it depended on the day, and the remainder noting it made them feel better. See “Appendix 3” for all quotes related to the following themes.

### Connection and acceptance

Participants believed the community helped them feel not alone; it helped to know that their struggles were normal. For instance, one participant noted, “the way what we go through is accepted without shame. we understand that our suffering manifests itself in strange ways and theres no embarrassment about behaviours, and thats helped me overcome a lot of guilt (P4),” while another user said, “The community helps break down the isolation that comes with having an ED (P25).” For some, humor made them feel connected: “i look at ironic/sarcastic posts about ed and feel understood (P6).”

In particular, the community helped some get support that they felt was missing in their daily lives. For example, one participant noted, “the fact that I can connect with other people going through recovery helps me feel less isolated. I can share how I'm feeling (I have trouble doing this face to face with people I know personally), which really helps me to understand my thinking and allows me to let off some steam (P24).”

One participant did note a negative outcome of the connection to the community, “i often feel obligated to offer help when i have barely enough love for myself, hindering my own progress (P4).” Similarly, another participant noticed that the acceptance could sometimes lead them to feel their disordered habits were not as concerning, “I think that as much as this community makes me feel validated, it also makes me feel like my eating disorder is somewhat of a normal thing… and I tend to see it as less serious than it is. Like 'Oh well I have bulimia, it's normal for me to binge and purge, nothing I can do about it (P6).'" Further, another participant noted, “I had a popular blog and got a lot of attention from that—which fed into my ed. otherwise nothing else (P26).” While the majority of participants found connection to be a positive part of the community, these participants indicated that that connection can have more negative outcomes as well. It is possible that how they experienced connection within the community may depend on which stage of the TTM participants were engaged in, as well as how enmeshed their overall identity still was with their eating disorder identity. In other words, connection to and acceptance from the community may have more positive outcomes for those engaged in the contemplation stage or more advanced stages of the model.

### Motivation and encouragement

Participants also felt that the community motivated them in their recovery, both generally from posts, and from seeing others succeed. For instance, one user noted that it helped, “Seeing so many people going on the right path (P28),” while another stated, “… one day, I could have the mindset of most of these people, who know that food and weight isn't everything (P10).” Finally, another participant stated that they could, “… do the right thing for myself because others are affirming that it IS the right thing (P11).”

Sometimes the encouragement involved direct advice: “… the advice that people who are recovered or who are further down the road than you are give help motivate you and help you through some of your struggles… (P14).” For others, reading about or listening to success stories was important, “The motivating speeches and pictures of people,who talk about for example spending their whole life fitting into a piece of fabric and then burning these clothes (P10).”

Finally, some participants found that seeing others struggle motivated them to focus more on their own recovery, as they wanted to avoid the difficulties they saw others experience; “It makes me see what a waste of a life starving/counting calories/obsessing is and how much more of a satisfying life people have without that. It shows me the real dangers of eating disorders such as serious/life threatening conditions. It shows that EDs are not a game, one must either recover or die of it (P12).”

It may be that such motivation and encouragement from others in the community assists users in battling the egosyntotic nature of their eating disorders, as it confirms and reinforces the idea that illness is not an achievement, and identity outside of their ED is possible. Motivation and encouragement may also help users transition through the TTM stages, particularly from contemplation through to action and maintenance.

### Content

Participants expressed mixed feelings about the content itself within the community. Some noted that whether or not they felt positive or distressed about their recovery, depended on the content posted at any given time. One participant said, “If I am having a bad day I will be less likely to fight my urges to engage in behaviors if I am triggered by something (P17),” while another said, “most people in the recovery tags on tumblr do not want to recover properly (P8).” Another participant described this theme well, nothing that it, “Depends on the content seem sometimes it's uplifting and other times it can make me upset (P18).”

Indeed, several participants noted feeling triggered by the presence of pro-ED content. One participant noted, “Some people post triggering posts, such as very low weights, or even things tagged in both pro recovery and pro ana… (P14),” while another commented on, “People in the #edrecovery tag who say about the calories they've eaten/are planning to eat… (P10).” One participant believed that the majority of the community is not actually interested in recovery, “… Most of the recovery community engages in and encourages extremely disordered behaviors like posting low weight photos, under eating, over exercising, etc. (P5),” while another noted, “There are alot of things out there to do with thinsporation that I haven't necessarily gone out of my way to find but have ended up on my news feed, which can cause me to begin a cycle of feeling bad about myself, and ultimately going to look for more thinspo (P13).” In these ways, content may feed into the egosyntonic nature of EDs, such that triggering posts and photos may reinforce the role of the illness in users’ identities.

However, others found the content encouraging, as it may be helpful to see, “… so many people going on the right path… seeing important reminders and affirmations (P26).” “It just provides inspirational quotes and pictures (P2),” and, “seeing important reminders and affirmations. Analysis of the behaviors in ways that help me realize where my anorexic behaviors stem from (issues that primarily effect women, parental control/abuse, LGBT stuff and other issues). It helps being able to understand where my problems stem from (P21).” It is possible that users’ experiences of content may depend on which stage of the TTM they would fall within; those that are further along in their recovery may seek out more recovery-specific content, and/or be better able to resist engaging with triggering content.

### Comparison and competition

Some participants also discussed a tendency among users to compare and/or compete with others in recovery; one participant noted, “The sense of competition has always been difficult for me in recovery settings… If one person says they engaged in a behavior, the voice in my head will scream ‘see, you are not strong like them’ (P17).” Others noted, “because its been hard for me to recover and I feel weak (P3),” and, “It makes me feel worse because I dislike not ‘winning’ at things… Also, when there seems to be kind of a severity ranking system… Some people seem to have, and they may be unaware of this or disagree with it on a factual level, kind of an idea that once you become weight restored or eat healthy that you're better and done, and therefore less sick than people who actively engage in behaviors (P19).”

However, some felt that seeing others’ struggles and successes made them feel more positively about recovery. For example, one participant noted, “I compare myself a lot but then I love seeing people do well, it inspires me (P14),” while another explained they felt positively because of the “… realization that (a) I don't want to be as sick as some others there (b) I can do the right thing for myself (P11).” The potential positive or negative effect of comparison and competition may be a particular result of the confluence between identification with EDs versus recovery, and the TTM. Specifically, those further along in the stages of the TTM may have experienced shifts in identity identification, such that those further in recovery identify more with symptom alleviation and overall health, rather than illness severity. Competition and comparison may in turn strengthen recovery identity, versus those earlier in the transitional stages who may find competition and comparison still invokes their identification with their ED.

## Discussion

This study qualitatively examined individuals’ perceptions of the ED recovery community on Tumblr, noting both positive and negative effects on recovery. Themes related to connection and acceptance, motivation and encouragement, content, and comparison and competition were identified by the authors. While participants expressed gratitude for the opportunities for social connection and recovery specific support, many struggled with triggering content, and more were ambivalent about the presence of comparison and competition within the community.

The theme of connection and acceptance was overall highly positive; participants expressed that they were able to feel validation and appreciation for their struggles. The community seemed to provide a sense of belonging and even relief for many who did not feel that they had that type of support outside of the community. Three participants did note that the connection could sometimes lead to pressure to give support, normalization of disordered habits, and that attention via connection to others could sometimes feed unhealthy habits. However, these findings overall support previous works on pro-recovery communities, such that feelings of connection and support were prevalent, even with the presence of triggering content (e.g., [1, 13, 14, 22]).

Indeed, participants frequently noted that content may be triggering; there was a sense by several that some people within the community purposefully posted triggering content under the guise of recovery. Others noted that the recovery hashtags often had content they perceived as pro-ED. These findings may be explained by the egosyntonic nature of EDs in that perhaps user posting such triggering content are not truly seeking recovery and do not see a problem with their disorder. The presence of pro-ED content may affect some participants more depending on their own internal states at the time of exposure, as some noted that the content many only affect them depending on their own current feelings about their recovery.

Such feelings about the content are related to the themes of comparison and competition, as well as motivation and encouragement. Many participants noted that the recovery community felt competitive; some commented on the desire to be the “best” at recovery, while others felt there was an unhealthy focus on severity of ED symptoms. However, some felt that seeing others struggle made them compare themselves positively, such that they wanted to make sure they did not experience those symptoms themselves. Broadly speaking, receiving positive advice and encouraging feedback, as well as viewing inspirational posts, was also very motivating for participants. These themes align with the more general findings of Saunders and Eaton [18], who found that engaging in comparisons to others resulted not only in recovery hindering thoughts and feelings, but also a similar number of recovery promoting beliefs. In particular, the theme of motivation and encouragement shows the possibility that participants may be in the contemplation stage of change as they are motivated to change from learning of the dangers of EDs which Cockell et al. [10] suggests is important for recovery.

This study extends these works by connecting with users to better understand their specific experiences, and how those may affect their internal states and recovery [4]. Participants suggest that they feel the recovery community is relatively beneficial; therefore, unmoderated communities may be another positive tool for those in the recovery process, especially as techniques to automatically identify particularly harmful and dangerous posts continued to be refined (e.g., [23]).

Beyond the overall examination of an unmoderated, online recovery community, a key strength of this study is the inclusion of open-ended questions, as participants could write about their lived experiences. The anonymity of the survey may have also allowed participants to feel comfortable expressing their genuine views. Further, these results add to the nuance of users’ experiences, providing a more in-depth picture of how individuals may benefit from unmoderated communities.

The findings from this study should be considered in light of the following limitations. First, our sample size may be considered small *(n* = 29), though we believe that the number of responses allowed us to gain a meaningful understanding of participants’ perspectives regarding the community [8]. In addition, the majority of participants were white females, which is not an accurate representation of individuals who develop EDs; our study’s demographics were similar to those found in previous studies, however (e.g., [17]). Future research may seek to determine why the dynamics of such communities deter more diverse populations from participating, and/or how to more responsibly engage diverse groups within these communities. Second, it is possible that participants were not fully representative of the recovery community on Tumblr. We tried to minimize this risk by posting commonly used hashtags in the recovery community to reach those actively involved. Further, the use of Tumblr has declined since the completion of this study. While we believe that these findings remain applicable to social media recovery communities more broadly, further research should explore new platforms. Additionally, while the goal was to minimize potential distress given the anonymity of the survey, we did not ask questions related to symptom severity or longevity; future studies may seek to do so. Lastly, the majority of our sample reported having AN or EDNOS/OSFED, which is not representative of the entire ED population. However, this pattern has also been found in related studies (e.g., [17]).

## Conclusion

As social media is ever-evolving, it is imperative to understand users’ experiences of online, unmoderated ED recovery communities. Given the barriers to ED treatment (e.g., [2]), free online recovery communities may be the main or only resource for some, or may be supplemental. This research suggests that, for most, these communities may provide an overall positive and encouraging experience, despite negative feelings around comparison and the presence of triggering content. Future research should continue to explore recovery communities on newer social media platforms, and seek to understand why such communities may not encourage the inclusion of diverse voices.

## Data Availability

The datasets used and/or analysed during the current study are available from the corresponding author on reasonable request.

## Appendix 1

### Original survey items

1. What is your age? ______

2. What is your gender? _______

3. Ethnicity: please check one of the following.

_____ Asian _____ Black _____ Hispanic _____ Native American _____ White ______ Biracial/multiracial _____ Other

4. Does the recovery community on Tumblr help you with **your eating disorder** recovery?

No    A little    Sometimes    Often    Most of the time

5. What about the recovery community helps your eating disorder recovery? (OPEN ENDED)

6. What about the recovery community do you think makes your eating disorder recovery harder? (OPEN ENDED)

7. Do you think that the information about recovering from eating disorders that is shared on Tumblr is accurate?

No    A little    Sometimes    Often    Most of the time    Optional-Explain (OPEN ENDED)

8. Have you found the eating disorder recovery community on Tumblr to be open and welcoming to all users?

No    Sometimes    Yes

9. Why or why not? (OPEN ENDED)

10. After viewing eating disorder recovery posts on Tumblr, do you usually feel better or worse about your own recovery? (OPEN ENDED)

11. Why? (OPEN ENDED)

12. What information have you learned about eating disorders or recovering from eating disorders **specifically from Tumblr?** (OPEN ENDED)

13–16. How engaged in the eating disorder recovery community on Tumblr are you? Check all that apply.

- Never reblog or like posts
- Reblog or like posts occasionally
- Reblog or like posts frequently
- Never create own posts
- Create own posts occasionally
- Create own posts frequently
- Never comment on others posts
- Occasionally comment on others posts
- Frequently comment on others posts
- Never message other members
- Occasionally message other members
- Frequently message other members

17. Have you been diagnosed (by a doctor?) with an eating disorder?

Yes    No

If yes, what have you been diagnosed with? (OPEN ENDED)

If no, what eating disorder do you think best describes what you are experiencing? (OPEN ENDED)

18. Are you receiving professional help to aid you in recovery?

Yes    No

## Appendix 2

### Additional likert-style question frequencies

**Table:**

Survey item	*N*, %
Help with recovery (*n* = 29)	
No	1 (3.4%)
A little	7 (24.1%)
Sometimes	7 (24.1%)
Often	10 (34.5%)
Most of the time	4 (13.8%)
Accuracy (*n* = 29)	
No	4 (13.8%)
A little	3 (10.3%)
Sometimes	10 (34.5%)
Often	10 (34.5%)
Most of the time	2 (6.9%)
Welcoming (*n* = 29)	
No	6 (20.7%)
Sometimes	9 (31.3%)
Yes	14 (48.3%)
Reblog (*n* = 28)	
Never	0 (0%)
Occasionally	16 (57.1%)
Frequently	12 (42.9%)
Create Posts (*n* = 28)	
Never	7 (25%)
Occasionally	18 (64.3%)
Frequently	3 (10.7%)
Comment (*n* = 28)	
Never	14 (50%)
Occasionally	11 (39.3%)
Frequently	3 (10.7%)
Message (*n* = 28)	
Never	9 (32.1%)
Occasionally	16 (57.1%)
Frequently	3 (10.7%)

“Help with recovery” refers to the question, “Does the recovery community on Tumblr help you with your eating disorder recovery?” “Accuracy” refers to the question, “Do you think that the information about recovering from eating disorders that is shared on Tumblr is accurate?” “Welcoming” refers to the question, “Have you found the eating disorder recovery community on Tumblr to be open and welcoming to all users?” “Reblog” refers to the frequency of reblogging or liking posts, “create posts” refers to how often participants created their own posts/content, “comment” refers to how often participants commented on other users’ posts, and “message” refers to how often participants message other users.

## Appendix 3

### Identified quotes related to overall study themes

**Table:**

**Theme: Connection/acceptance**
• “It's nice to know that *I am not alone* in my disorder or my feelings (P1).” • “*I feel less alone* (P1).” • “It just provides inspirational quotes and pictures and *helps me realize that im not the only one going through this* (P2).” • “the way *what we go through is accepted without shame. we understand that our suffering manifests itself in strange ways and theres no embarrassment about behaviours*, and thats helped me overcome a lot of guilt (P4).” • “i often feel *obligated to offer help* when i have barely enough love for myself, hindering my own progress (P4).” • “it helps me *make me feel less alone*. i look at ironic/sarcastic posts about ed and *feel understood and validated* (P6).” • “think that *as much as this community makes me feel validated, it also makes me feel like my eating disorder is somewhat of a normal thing. I start seeing my disorder as a "common" thing*, and I tend to see it as less serious than it is. Like "Oh well I have bulimia, it's normal for me to binge and purge, nothing I can do about it (P6)." • “It *lets me know that my thoughts aren't uncommon* among those with EDs and *it helps me recognise that my ED is valid and very real* (P7).” • “*It's reassuring to find others who experience things the same way you do* (P8).” • “There are *a lot of people on the same journey as i am* (P9).” • “Provides… *affirmation that difficulties are valid* (P11).” • “It *helps to know that others go through the same thing* although pretty much everyone is twenty years younger than me (P12)!” • “*Connecting and sharing with people who understand you and what you are going through*… People in the recovery community *know what you are going through* and are very well placed to give advice (P14).” • “As people are *going through the same thing as me and I am not alone* (P15).” • “It *allows me to feel validated* (P16).” • “It makes *me feel like I am not alone in my struggles, and the things I experience are not out of the ordinary. It makes me feel less crazy* (P17).” • “… Also *feel like your not the only one* (P18).” • “Because it's *less isolating, and because you can see that people are struggling too and that it's okay…* Another reason is that being thin is very valued in the West, and when *you communicate with people in an environment who are now encouraging themselves and others to reject that, it creates more positive feelings towards recovery* (P19).” • “There are motivational posts and *people I can relate to. It helps me not feel alone* (P23).” • “the fact that I can *connect with other people going through recovery helps me feel less isolated. I can share how I'm feeling (I have trouble doing this face to face with people I know personally), which really helps me to understand my thinking and allows me to let off some steam* (P24).” • “Having *contact with people who have similar experiences and feelings as I do. The community helps break down the isolation* that comes with having an ED (P25).” • “… *not feeling like I'm alone, having people relate to what I'm going through when no one in real life can* (P26).” • “I had a popular blog and *got a lot of attention from that—which fed into my ed* (P26).” • “… reminds me what I'm fighting for and that *I'm not alone* (P26).” • “… *knowing that I can help others who might be struggling* (P28).”
**Theme: Motivation/Encouragement**
• “… read analyses and articles so that I can *better understand my condition and reflect upon it* (P6).” • “It also lets me see other peoples progress which *inspires me to do the same because I have seen that it is possible to recover* (P7).” • “… if the content is educational with well written and recovery oriented articles or even posts about how they fought their eating disorder that *day I will find myself coming out with a piece of knowledge or a little bit of hope that I didn't have before* (P7).” • “*Encouragement from others* (P9).” • “*Because I know that there is still hope for me and that one day, I could have the mindset of most of these people, who know that food and weight isn't everything that's important*. These are things you shouldn't think much about (P10).” • “Provides *encouragement to do the right thing…* (P11).” • “*I can do the right thing for myself because others are affirming that it IS the right thing* (P11).” • “*It makes me see what a waste of a life starving/counting calories/obsessing is and how much more of a satisfying life people have without that. It shows me the real dangers of eating disorders such as serious/life threatening conditions. It shows that EDs are not a game, one must either recover or die of it*… (P12)” • “*Seeing people succeed and the advice that people who are recovered or who are further down the road than you are give help motivate you and help you through some of your struggles* because let's face it most professionals have no clue. People in the recovery community know what you are going through and *are very well placed to give advice* (P14).” • “*Staying strong and positive* (P15).” • “*The mot aviation of others and the willingness of them to help others completely* (P16).” • “*Get counseling advice* and recovery information (P18).” • “Other bloggers can also be very kind *and leave positive and encouraging messages* (P19).” • “It *helps me staying motivated* (P20).” • “Because it reminds me of what I really wants (P20).” • “*Motivation* (P22).” • “*There are motivational posts* and people I can relate to (P23).” • “*I know that I am making progress* outside of what i post from tumblr (P23).” • “*Support* (P27).”
**Theme: Content**
• “Sometimes, *hearing about negative experiences* makes me dwell on my own (P1).” • “It just *provides inspirational quotes and pictures* (P2)” • “I think that it can sometimes be triggering to see *others post low body weight photos or pictures of food that they know are restricted* (P2).” • “I really like *reading quotes and poems about recovery*- i usually print them out and hang them on my wall (P2).” • “i am at a stable, healthy point. however when im vulnerable i find that *the wrong words can make me contemplate relapse, but the right post can be the reason i eat dinner* (P4).” • “Most of the recovery community engages in and encourages extremely disordered behaviors like *posting low weight photos, under eating, over exercising, *etc.(P5).” • “i look at *ironic/sarcastic posts* about ed and feel understood and validated, or *read analyses and articles*… (P6).” • “It also lets me *see other peoples progress* which inspires me to do the same because I have seen that it is possible to recover (P7).” • “… *Seeing others with eating disorders that are thinner than I am, are inpatient or have ng tubes* tells me that because i'm not 'that sick' it doesn't matter (P7).” • “Obviously *content is different day to day and if one day there is a strong presence of triggering posts such as those including calorie counting and weight gains/losses or before and after photos* I am more likely to come out feeling negative. However, *if the content is educational with well written and recovery oriented articles or even posts about how they fought their eating disorder that day* I will find myself coming out with a piece of knowledge or a little bit of hope that I didn't have before (P7).” • “… *The oversharing of triggering things and body checks and restricted portions of food* coupled with the orthorexic obsession with being "healthy" and eating "clean" makes it so much harder to recover or to see what would be truely healthy and I think it's very damaging (P8).” • “*Triggering numbers regarding the weight of others* (P9).” • “The *motivating speeches and pictures of people*, who talk about for example spending their whole life fitting into a piece of fabric and then burning these clothes. *Pictures of women considered 'fat' who are very body-positive* and name the positives of recovery (P10).” • “People in the *#edrecovery tag who say about the calories they've eaten/are planning to eat, binge or purge photos or even talking about it, pictures of food* (P10).” • “When people with a recovery blog get to a bmi of say 18–20 then relapse. *People posting pictures of food especially when it's not even food they are eating, if it is their lunch that is kind of cool and helpful but if it is just food porn and they're not really eating*…*then they aren't recovered? When people claim to be recovered but obsess about healthy food and working out or veganism or fad diet trends. Someone posted a photo of herself wearing the jeans she wore to the clinic at the beginning of her recovery and those jeans were two or three sizes bigger*…*so before and after recovery pics where she is slimmer now due to eating vegan. Selfies of people in recovery or recovered where they are still at a low bmi and all the compliments of how gorgeous they look*…that is all triggering (P12).” • “I like *simple reminders to eat* (P12).” • “There are *alot of things out there to do with thinsporation that I haven't necessarily gone out of my way to find but have ended up on my news feed*, which can cause me to begin a cycle of feeling bad about myself, and *ultimately going to look for more thinspo* (P13).” • “*Seeing people succeed and the advice* that people who are recovered or who are further down the road than you… (P14)” • “Some people post *triggering posts, such as very low weights, or even things tagged in both pro recovery and pro ana*, which is very annoying (P14).” • “*Triggered photos or comments* (P15).” • “The *low weight/Calorie intake posts* can be triggering sometimes (P16).” • “If I am having a bad day I will be less likely to fight my urges to engage in behaviors if I *am triggered by something* (P17).” • “Get counseling advice and *recovery information…* (P18).” • “*Pro Ana and pro Mia content* and it's hard to look away when you see that (P18).” • “*Depends on the content seem sometimes it's uplifting and other times it can make me upset* (P18).” • “*People talking about numbers*… Also, veganism. *It's hard to read people critique ways of eating and create an awareness of food in a negative way*. If you need to eat meat in your recovery, having someone say that eating meat is evil can be a little detrimental (P19).” • “*Analysis of the behaviors in ways that help me realize where my anorexic behaviors stem from (issues that primarily effect women, parental control/abuse, LGBT stuff and other issues).* It helps being able to understand where my problems stem from (P21).” • “People that still engage in *public discourse on tumblr that is harmful or encouraging of the eating disorder*. Even *girls who know better and say that they are against pro-Ana will still post pictures of themselves at low weights or triggering material* (P21).” • “*Possible Triggers* (P22).” • “There are *motivational posts* and people I can relate to (P23).” • “There are some people who *post triggering content on the recovery pages (such as thinspo, numbers, or behaviors)* (P23).” • “*pro-anorexia/eating disorder pages*. I feel the pressure from these blogs to step back into my old habits (P24).” • “*Photos of individual's meals. Posts about low weights* (P25).” • “*Posts that provide links to resources and articles have been helpful. Personal posts that avoid using specific information that may be triggering help me feel less alone. On the flip side, posts with triggering information (caloric intake, weight, *etc*.) can be harmful* (P25).” • “… seeing *important reminders and affirmations* (P26).” • “*Thinspo *etc.(P27).” • “*Seeing so many people going on the right path*… (P28).” • “Recovery in general is hard, but *when people post "body checks*" it seems counterproductive (P28).” • “*People seeming to be happy and find their health* (P29).”
**Theme: Comparison/Competition**
• “Sometimes, *hearing about negative experiences makes me dwell on my own* (P1).” •* “I am a highly competitive type A personality*. Unfortunately one of the lies my ED tells me is that I'm not sick enough, that my ED isn't real because I'm not at a dangerously low weight. *Seeing others with eating disorders that are thinner than I am, are inpatient or have ng tubes tells me that because i'm not 'that sick' it doesn't matter. Thus, it feeds the ED thoughts and allows them to continue and fester so that they take over more and more of my brain* (P7).” •* “Sometimes triggering when others are doing worse, or when they can access higher levels of care and support that aren't available personally…* (P11).” •* “Comparison is still there but that is not the community's fault, it's just the nature of the disorder* (P14)*.”* •* “The sense of competition has always been difficult for me in recovery settings. Even throughout treatment I struggled with the competitive nature of eating disorders. If one person says they engaged in a behavior, the voice in my head will scream ‘see, you are not strong like them.’* It is all about thought challenging, and recognizing that the voice in your head is the devil on your shoulder (P17).” • “People talking about numbers*. It still seems a little competitive. Also, when there seems to be kind of a severity ranking system,* or when there is, I'm not sure I'll formulate this idea correctly, an innocuous dismissal of sufferers who may not have "traditional" EDs. Some people seem to have, and they may be unaware of this or disagree with it on a factual level, kind of an idea that once you become weight restored or eat healthy that you're better and done, and therefore less sick than people who actively engage in behaviors (P19).” • “Since I've gained weight *I feel that I'm ‘not skinny enough’ to have an eating disorder*, and that has been triggering for me (P24).” • “*When people look better then me* (P29).*”* • *“because its been hard for me to recover and i feel weak* (P3).*”* • “realization that a) I don't want to be as sick as some others there… (P11).” • “*I compare myself a lot* but then I love seeing people do well, it inspires me (P14).” • “because it can show me *how I'm not really committing myself to recovery, or how I'm not trying very hard, or still engaging in behaviors that I considered to be normal and acceptable. It makes me feel worse because I dislike not "winning" at things* (P19)*.”* •* “I seem to be making faster progress than some others*, in terms of weight regain*, because they seem to find it harder* to regain weight and healthy eating habits (P24).*”* •* “Makes me compare myself to them* (P27)*.”*
